# Neutralizing monoclonal antibodies protect against human adenovirus type 55 infection in transgenic mice and tree shrews

**DOI:** 10.1080/22221751.2024.2307513

**Published:** 2024-01-19

**Authors:** Xinglong Liu, Zhengfeng Li, Xiao Li, Xiaoyan Zhang, Yali Zheng, Wan Su, Ying Feng, Yutong Liu, Weixuan Wu, Xikui Sun, Nana Wang, Xianmiao Ye, Zhichao Zhou, Wenkuan Liu, Jun He, Wei Wang, Linbing Qu, Rong Zhou, Ling Chen, Liqiang Feng

**Affiliations:** a State Key Laboratory of Respiratory Disease, CAS Key Laboratory of Regenerative Biology, Guangzhou Institutes of Biomedicine and Health, Chinese Academy of Sciences, Guangzhou, People’s Republic of China; b University of Chinese Academy of Sciences, Beijing, People’s Republic of China; c State Key Laboratory of Respiratory Disease, National Clinical Research Center for Respiratory Disease, Guangzhou Institute of Respiratory Health, the First Affiliated Hospital of Guangzhou Medical University, Guangzhou Medical University, Guangzhou, People’s Republic of China; d Guangzhou Laboratory & Bioland Laboratory, Guangzhou, People’s Republic of China; e Guangzhou nBiomed Ltd., Guangzhou, People’s Republic of China

**Keywords:** Human adenovirus type 55; neutralizing monoclonal antibodies; rhesus macaque; targeting sites; mechanism of action

## Abstract

Re-emerging human adenovirus type 55 (HAdV55) has become a significant threat to public health due to its widespread circulation and the association with severe pneumonia, but an effective anti-HAdV55 agent remains unavailable. Herein, we report the generation of macaque-derived, human-like monoclonal antibodies (mAbs) protecting against HAdV55 infection with high potency. Using fluorophore-labelled HAdV55 virions as probes, we isolated specific memory B cells from rhesus macaques (*Macaca mulatta*) that were immunized twice with an experimental vaccine based on E1-, E3-deleted, replication-incompetent HAdV55. We cloned a total of 19 neutralizing mAbs, nine of which showed half-maximal inhibitory concentrations below 1.0 ng/ml. These mAbs recognized the hyper-variable-region (HVR) 1, 2, or 7 of viral hexon protein, or the fibre knob. In transgenic mice expressing human desmoglein-2, the major cellular receptor for HAdV55, a single intraperitoneal injection with hexon-targeting mAbs efficiently prevented HAdV55 infection, and mAb 29C12 showed protection at a dose as low as 0.004 mg/kg. Fibre-targeting mAb 28E8, however, showed protection only at a dose up to 12.5 mg/kg. In tree shrews that are permissive for HAdV55 infection and disease, mAb 29C12 effectively prevented HAdV55-caused pneumonia. Further analysis revealed that fibre-targeting mAbs blocked the attachment of HAdV55 to host cells, whereas hexon-targeting mAbs, regardless of their targeting HVRs, mainly functioned at post-attachment stage via inhibiting viral endosomal escape. Our results indicate that hexon-targeting mAbs have great anti-HAdV55 activities and warrant pre-clinical and clinical evaluation.

## Introduction

Human adenovirus type 55 (HAdV55), initially isolated in 1969 and termed as HAdV11a [1], is a non-enveloped, double-stranded DNA virus belonging to family *Adenoviridae*, species B. During recent years, HAdV55 has become one of the leading respiratory pathogens in China and South Korea [2-7]. Among the 70 reported HAdV outbreaks from 2009 to 2020 in China, HAdV55 caused 16 ones, second only to HAdV7 [4]. In South Korea, HAdV55 has caused several large outbreaks in the military since 2014, and is the most prevalent type among HAdV infections [6]. Unlike HAdV3, HAdV4 and HAdV7 which cause severe respiratory diseases mostly in young children, the elderly, and immuno-compromised patients, HAdV55 seems to cause fatal pneumonia in both children and immuno-competent young adults [8–10]. Severe HAdV55 infections have been documented in Turkey, Israel, France, China, and South Korea [3, 5, 8–16]. Clinical manifestations include high fever, cough, respiratory failure, and bilateral consolidations [12]. A proportion of critically ill patients still die despite receiving mechanical ventilation or even extracorporeal membrane oxygenation [16]. Currently, there is no specific anti-HAdV55 treatments. Several broad-spectrum antiviral drugs, such as ribavirin, ganciclovir and cidofovir, have been employed to treat severe HAdV infection in immuno-compromised patients, but they are not commonly recommended for immuno-competent ones due to limited antiviral efficacy or side effects such as nephrotoxicity [17]. A safe and effective antiviral agent against HAdV55 infection, therefore, is urgently needed.

Monoclonal antibodies (mAbs) are promising in combating severe viral diseases such as those caused by Ebola virus, respiratory syncytial virus, and SARS-CoV-2 [18, 19]. As for HAdV55 infection, neutralizing mAbs have the potential to directly block viral entry and to promote viral clearance via Fc-mediated effector functions [18], and thus suppress infection and prevent disease progression. Similar to other HAdVs, HAdV55 has an icosahedral capsid consisting of three major proteins (fibre, penton base, and hexon) and four minor/cement proteins (IIIa, VI, VIII, and IX). Fibre protrudes from each vertex and mediates viral attachment to the primary cellular receptor, human desmoglein-2 (hDSG2) [20]. Penton base forms the twelve vertices and triggers endocytosis after binding to the co-receptor integrin [21]. Hexon, the most abundant capsid protein, recruits dynein for transport to the nucleus at post-endosomal stage and mediates the docking of capsid onto nuclear pore complexes [22]. Minor capsid proteins are important for viral assembly, disassembly, endosome penetration and capsid stabilization [23]. It has been reported that fibre and hexon, in particular the knob domain of fibre and the hyper-variable-regions (HVRs) on the surface of hexon, are the major targets of anti-HAdV neutralizing antibodies (nAbs) [24–26]. Fibre-targeting nAbs block the binding of HAdVs in cell cultures, but in animal models or humans their protective effects remain elusive [27, 28]. Hexon-targeting nAbs unlikely block viral binding, but rather inhibit endosomal escape or microtubule-dependent transport toward nucleus [29–31]. HAdV55 is an inter-typic recombinant of HAdV11 and HAdV14, another two HAdVs associated with renal and respiratory diseases respectively [32]. HAdV55 contains a partial hexon gene of HAdV11 and the backbone of HAdV14, and shares similar but somewhat different hexon and fibre proteins with HAdV11 and HAdV14, respectively [32]. Anti-HAdV55 nAbs may also neutralize HAdV11 or HAdV14, depending on their targeting sites.

There are difficulties for generating human-derived, high-potent anti-HAdV55 nAbs. It is challengeable to find ideal blood donors with high titres of nAbs, due to the low seroprevalence in civilian populations [33, 34]. Several groups have generated nAbs using mice immunized with inactivated HAdV55 or purified fibre knob protein [27, 28]. However, clinical employment of murine antibodies usually requires iterative and time-consuming humanization [18]. It remains unclear whether the obtained fibre-targeting nAbs have *in vivo* protective effects, although they had *in vitro* neutralizing activities to HAdV55 and even to HAdV7 or HAdV11 [27, 28]. In addition, fibre-targeting nAbs might have much lower potency than hexon-targeting ones [35]. An attractive alternative approach is to generate nAbs from rhesus macaques (*macaca mulatta*) that have been immunized with experimental vaccines. It has been reported that rhesus macaques share over 92% homology in the immunoglobulin (Ig) genes with humans [36], and a human-macaque chimeric strategy further improves the homology [37]. In the context of repeated immunization, memory B cells can undergo multiple rounds of maturation, promoting the occurrence of high-potent nAbs [36, 37]. Through this approach, we have successfully generated human-like nAbs against high virulent H5N1 influenza virus and Ebola virus, which showed extremely high neutralizing potency both *in vitro* and *in vivo* [37, 38].

In this study, we generated human-like, monoclonal anti-HAdV55 nAbs using rhesus macaques that had been immunized twice with an E1- and E3-deleted, replication-incompetent HAdV55. We isolated specific memory B cells using fluorophore-labelled HAdV55 virions, cloned the coding sequences for the variable regions of IgG heavy (VH) and light (VL) chains via single-cell PCR technology, and constructed full-length nAbs using the constant regions of human IgG1. Based on our previously reported HAdV14-HAdV55 hexon-chimeric viruses [26], we identified the targeting sites of each nAb. Importantly, we comprehensively tested the protective effects of these nAbs in hDSG2 transgenic mice and tree shrews [20, 39]. We also dissected the mechanism of action of representative nAbs with distinct targeting epitopes.

## Materials and methods

### Cells and viruses

Human embryonic kidney 293 (HEK293, No. CRL-1573), HEK293T (No. CRL-3216) and Human lung carcinoma A549 (No. CCL-185) cells were purchased from American Type Culture Collection (ATCC) and cultured in DMEM with 10% fetal bovine serum (FBS). Expi293F cells were purchased from Thermo Fisher Scientific (R79007) and cultured in Union 293 Cell Feed Medium (Union-Biotech) with 10% FBS. All cells were incubated at 37°C in an atmosphere containing 5% CO_2_.

HAdV55 Shanxi-Y16 strain (GenBank No. KF911353.1), HAdV14p1 GZ01 strain (GenBank No. JQ824845.1), recombinant HAdV55 and HAdV14 expressing enhanced green fluorescent protein (EGFP) or secreted alkaline phosphatase (SEAP), and chimeric HAdV14 viruses harbouring each of the 7 HAdV55 HVRs have been described previously [26]. HAdV11 Slobitski strain (GenBank No. AF532578.1) was purchased from ATCC and propagated in A549 cells. Recombinant HAdV11 expressing EGFP or SEAP were constructed as the following. In brief, HAdV11 viral particles were treated with sodium-dodecyl-sulfonate (SDS) and the genomic DNA was extracted using phenol–chloroform. The terminal regions were amplified by PCR and sub-cloned into pMD18T (TaKaRa) to obtain a shuttle plasmid. Genomic plasmid pAd11 was constructed by homologous recombination between linearized shuttle plasmid and viral genomes in *E.coli* BJ5183 competent cells (Agilent Technologies). The fragments flanking the E3 region were amplified by PCR and sub-cloned into pVAX1 plasmid (Thermo Fisher Scientific) to obtain p11E3LR. Viral E3 region was then deleted by homologous recombination between E3LR and linearized pAd11 to obtain pAd11ΔE3. The coding sequences for EGFP and SEAP, flanked by a CMV promoter and a BGH poly(A) signal, were separately inserted into p11E3LR to obtain shuttle reporter plasmids. pAd11-EGFP and pAd11-SEAP were then constructed by homologous recombination between shuttle reporter plasmids and linearized pAd11ΔE3. Finally, HAdV11-EGFP and HAdV11-SEAP were rescued by releasing the viral genomes from pAd11-EGFP and pAd11-SEAP and transfecting into HEK293 cells. HAdV55ΔE1ΔE3 was constructed based on previously reported HAdV55ΔE3 [26]. The E1 and E4 regions of pAd55ΔE3 were deleted by homologous recombination to obtain pHAdV55ΔE1ΔE3ΔE4. HAdV5 E4 gene was amplified by PCR and inserted into the original E4 region of pHAdV55ΔE1ΔE3ΔE4 to obtain pHAdV55ΔE1ΔE3. The genomic DNA was released from pHAdV55ΔE1ΔE3 by enzymatic restriction digestion and transfected into HEK293 cells to rescue HAdV55ΔE1ΔE3. Wild-type HAdV55, HAdV11, HAdV14, HAdV55ΔE1ΔE3, and the reporter viruses were all propagated in HEK293 cells and purified by caesium chloride gradient centrifugation. Infectious virions were titrated and recorded as median tissue culture infectious dose (TCID_50_) as described [26]. Virus stocks were stored at −80°C.

### Animals

hDSG2 transgenic mice have been described elsewhere [20]. Rhesus macaques (4-year-old, female, 3–5 kg) were purchased from Guangdong Landau Biotechnology Co. Ltd. All the macaques have been confirmed free of simian retrovirus, simian immunodeficiency virus or simian T lymphotropic virus type 1, and negative for nAbs against HAdV55, HAdV11, or HAdV14. Mouse and macaque experiments were carried out at the Animal Center of Guangzhou Institutes of Biomedicine and Health (GIBH) and were approved by the Institutional Animal Care and Use Committee (IACUC) of GIBH (Nos. 2018027 and 2018042, respectively). Tree shrews were purchased from the Animal Experimental Centre of Kunming Medical University (approval No: SCXK-K2020-0004) and acclimated under standard laboratory conditions. All the tree shrew experiments were in accordance with the “Guiding Principles in the Care and Use of Animals (China)” and were approved by the Laboratory Animal Ethics Committee of Affiliated First Hospital of Guangzhou Medical University (GMU).

### Immunization of rhesus macaques

Two macaques were intramuscularly (i.m.) inoculated with HAdV55ΔE1ΔE3 at 1 × 10^11^ viral particles (v.p.) in 1 ml phosphate-buffered saline (PBS) twice at a 32-week interval. The other two macaques were injected with an equal volume of PBS at the same time points. At 1, 2, 3 or 4 weeks after the second immunization, serum samples were collected and subjected to neutralization assay. Peripheral blood mononuclear cells (PBMCs) were isolated by OptiPrep density gradient separation (Serumwerk Bernburg) and subjected to memory B cell sorting.

### HAdV55ΔE1ΔE3 labelling

Purified HAdV55ΔE1ΔE3 virions were labelled with Alexa Fluor 647 N-hydroxysuccinimide (NHS) ester (Thermo Fisher Scientific) according to manufacturer’s guidelines. In brief, 1 × 10^11^ v.p. of HAdV55ΔE1ΔE3 was added with 0.2 M Alexa Fluor 647 NHS ester in PBS at 4°C for 2 h. After coupling, unbound dye was removed by separation on Amicon Ultra Centrifugal Filters (Millipore). For confirmation, the labelled and unlabelled virions were separately added onto confluent A549 cells (1 × 10^4^ v.p. per cell). After incubation for 2 h, cells (ratios of cells infected by labelled virions versus those by unlabelled virions: 100: 0, 70: 30, 30: 70, 0: 100, respectively) were washed and analysed by Accur C6 Plus Flow Cytometer (BD Biosciences).

### Single B cell sorting, single-cell PCR, sequencing, and plasmid cloning

Freshly isolated macaque PBMCs were stained with the following antibodies at 4°C for 30 min in the dark: PE mouse anti-human CD3 (RRID: AB_394342), PE mouse anti-human CD14 (RRID: AB_314188); PE mouse anti-human CD56 (RRID: AB_395906); PE/Cyanine7 mouse anti-human CD20 (RRID: AB_314260); BB700 mouse anti-human CD27 (RRID: AB_2739731); APC-H7 mouse anti-human IgG (RRID: AB_10611877). Subsequently, PBMCs were incubated with Alexa Fluor 647-labelled HAdV55 virions at 4°C for 1 h in the dark. Dead cells were stained by the LIVE/DEAD Fixable Aqua Dead Cell Stain Kit (Thermo Fisher Scientific). HAdV55-specific memory B cells were designated as CD3^-^, CD14^-^, CD56^-^, CD20^+^, CD27^+^, IgG^+^ and HAdV55^+^. Individual B cells were sorted into 96-well plates containing lysis buffer using FACS Aria II flow cytometer (BD Biosciences). PCR plates were frozen on dry ice and stored at −80°C, or immediately subjected to reverse transcription (RT) [40].

The coding sequences for VH and VL chains were amplified by nested PCR according to a previously described protocol [40]. In brief, the cDNAs for VH and VL were obtained by RT using GoScript Reverse Transcription System (Promega). First-round PCR was performed using KOD FX Neo (Toyobo) and primers corresponding to macaque Ig genes [40]. The programme for the first round PCR: 95°C for 5 min; 50 cycles of 98°C for 10 s, 64°C to 52°C for 45 s (decreased 3°C per 5 cycles in the first 20 cycles), and 68°C for 1 min; 68°C for 10 min before cooling to 4°C. The programme for the second round PCR: 95°C for 5 min; 50 cycles of 98°C for 10 s, 60°C for 45 s, and 68°C for 1 min; 68°C for 10 min before cooling to 4°C. PCR products were analysed on 2.0% agarose gels, purified using HiPure Gel Pure DNA Mini Kit (Magen), and subjected to Sanger sequencing. Sequences were analysed using IMGT/V-QUEST (http://www.imgt.org/) [41, 42]. Paired VH and VL fragments were cloned into human IgG1 framework harboured in pCI-neo vector using ClonExpress II One Step Cloning Kit (Vazyme). Full-length macaque-human chimeric mAbs were expressed by co-transfecting HEK293T or Expi293F cells with equal amounts of paired heavy and light chain plasmids. Culture media were harvested on day 3 (HEK293T) or 5 (Expi293F) after transfection. Antibodies were purified by affinity chromatography using HiTrap Protein A HP (Cytiva).

### Enzyme-linked immunosorbent assay (ELISA)

In brief, purified HAdV55 viral particles were treated with 10% SDS. Fibre knob (residues 118–325) were produced and purified as described previously [26]. ELISA plates were coated with 100 ng per well viral lysates or fibre knob at 4°C overnight, and blocked with PBS containing 5% skimmed milk and 0.05% Tween 20 (PBST) at room temperature for 2 h. After washed with PBST, the plates were added with culture supernatants (100 μl per well) or purified mAbs (100 ng per well) and incubated at 37°C for 2 h. Subsequently, the plates were added with horseradish peroxidase (HRP)-conjugated goat anti-mouse antibodies (Beyotime Biotechnology) and incubated at 37°C for 1 h. After washed with PBST, the plates were added with chromogenic enzyme substrate. The reaction was stopped with 2 M H_2_SO_4_ and the optical density (O.D.) at 450 nm was measured with Microplate Reader Epoch2 (BioTek).

### Neutralization assay

In brief, HEK293 cells were seeded into 96-well plates at 2 × 10^4^ cells per well. Two days later, serial dilutions of macaque sera or mAbs were incubated with HAdV55-SEAP, HAdV11-SEAP, or HAdV14-SEAP at 2 × 10^6^ v.p. per well at 37°C for 1 h. The mixtures were added to the plates and incubated at 37°C for 24 h. Finally, the culture supernatants were harvested and SEAP activities were measured using the Phospha-Light System (Thermo Fisher Scientific). Relative light units (RLU) were recorded by GloMax Discover Microplate Reader (Promega). The neutralizing titres of immune sera were calculated as the reciprocal of the dilutions at which 50% of RLU values were inhibited. The IC50 values of mAbs were calculated similarly.

mAb targeting sites were analysed by neutralization assay based on HAdV55-EGFP, HAdV11-EGFP, HAdV14-EGFP, as well as chimeric HAdV14-EGFP. Each mAb was diluted to 100 ng/ml and incubated with each virus (2 × 10^6^ v.p. per well) at 37°C for 1 h. The mixtures were added to A549 cells in 96-well plates. After incubation at 37°C for 24 h, cells were fixed in 10% neutral buffered formalin (NBF) for 10 min. EGFP signals were excited at 488 nm and detected at 509 nm by Multimode Reader Mithras2 LB 943 (Berthold) and recorded as RLU. The neutralizing activities of the mAbs against each HAdV were calculated as Neutralization % = (RLU_virus_-RLU_mAb._)/ RLU_virus _× 100%.

### Western-blotting assay

In brief, HAdV55 lysates were separated by SDS-PAGE in the presence or absence of β-mercaptoethanol (β-ME). Proteins were transferred onto nitrocellulose filter membranes. After blocked with 5% BSA in PBST, membranes were incubated with each mAb (28E10, 28A5, 28E8, 29C12, 28F3) at 1 μg/ml at 4°C overnight. Finally, membranes were incubated with a HRP-conjugated goat anti-human antibody (Beyotime) and developed with StarSignal Chemiluminescent Assay Kit (GeneStar).

### Protective efficacy of mAbs in hDSG2 transgenic mice

In brief, 6–8 weeks old female hDSG2 mice (5 per group) were i.p. injected with each mAb. For comparison of the mAbs, the dose of each mAb was 0.5 mg/kg in 100 μl PBS. For comprehensively assessing the efficacy, the dose of mAb 29C12 was 0.5, 0.1, 0.02, or 0.004 mg/kg, and the dose of mAb 28E8 was 12.5, 2.5, or 0.5 mg/kg. Mice receiving an equal volume of PBS were used as controls. One day later, the mice were i.v. infected with 2 × 10^10^ v.p. of HAdV55-SEAP. Serum samples were collected at 1, 3 and 5 days after challenge and SEAP activities were measured. At 5 days after the challenge, mice were sacrificed and liver tissues were subjected to viral load detection.

The protective effects of each mAb against HAdV11 and HAdV14 infection were assessed similarly using hDSG2 mice. The doses of mAbs 28E10, 28A5, 29C12, and 28F3 were 0.5 mg/kg, and that of mAb 28E8 was 12.5 mg/kg. Serum samples and liver tissues were collected at 1 and 5 days after challenge, respectively.

### Protective efficacy of mAb 29C12 in tree shrews

In brief, 6–8 months old tree shrews (1 male and 3 female in each group) were i.p. injected with 10 or 2.5 mg/kg of mAb 29C12, or 10 mg/kg of a Zika virus-specific mAb 8D10 (human IgG1) as an irrelevant control [43]. One day later, tree shrews were intranasally (i.n.) infected with 5 × 10^6^ TCID_50_ of HAdV55 in 100 μl PBS. Healthy control groups were given with an equal volume of PBS. At 5 days after challenge, tree shrews were sacrificed, and the turbinate bone, trachea and lung tissues were collected for viral load titration. Lung tissues were also subjected to histopathological analysis.

### qPCR

Genomic DNA of HAdV55, HAdV11, and HAdV14 in cells or tissues was extracted using QIAamp DNA Mini Kit (Qiagen) and subjected to qPCR using iTaq Universal SYBR green Supermix (Bio-Rad). The primers were as follows: Q-Ad F, 5’- CATGGT GTGGAAGATGAACTTCC-3’, and Q-Ad R, 5’-GGAAACTTCGCCATAGATTG GC-3’. The programme was set up as 95°C for 5 min; 45 cycles of 95°C for 30 s, 55°C for 30 s, and 72°C for 30 s; and a melting curve was produced at 65°C to 95°C with an increment of 0.5°C per cycle for 5 s. Genome copies were calculated by the standard curves made using serially diluted genomic DNA extracted from viral stocks.

### Histopathology

Tree shrew lung tissues were fixed in 10% NBF for 24 h. Paraffin-embedded tissue sections were stained with haematoxylin and eosin (H&E), scanned, and assessed by a board-certified pathologist in a single-blind manner. Lymphocyte infiltration, bronchial epithelial hyperplasia, epithelial bronchioles, alveolar cavity dilatation, and interstitial thickening were graded as 0 (absence), and 1 (presence). The score of one section is calculated as the sum of the scores from five random fields of version.

### Viral binding and entry assays

The binding and entry assays were performed according to a previously described method [20]. In brief, HAdV55ΔE1ΔE3 (1 × 10^4^ v.p. per cell) was pre-incubated with mAb 28E10, 28A5, 29C12, 28F3, 28E8, or the irrelevant mAb 8D10 at 1 μg/ml at 37 °C for 2 h. After chilled on ice, the mixtures were added to A549 cells. For the binding assay, cells were incubated at 4°C for 2 h, washed with ice-cold PBS and subjected to DNA extraction. For the entry assay, cells were incubated at 37°C for 2 h, thoroughly washed with PBS and subjected to DNA extraction. The genome copies of cell-bound or internalized HAdV55 were determined by qPCR.

### Early endosome, cytoplasm, nucleus isolation, and confocal imaging

HAdV55ΔE1ΔE3 (1 × 10^4^ v.p. per cell) were incubated with mAb 28E10, 28A5, 29C12, 28F3, 28E8, or mAb 8D10 at 1 μg/ml at 37°C for 2 h. A549 cells were infected with the mixtures at 37°C for 0, 2, 12, 24 h and then washed twice with ice-cold PBS. Early endosome, cytoplasm, and nucleus were isolated using the Minute™ Endosome Isolation kit (Invent) following manufactory’s instructions. Viral DNA was extracted from each component and subjected to qPCR.

For confocal imaging, Alexa Fluor 647-labelled HAdV55ΔE1ΔE3 were incubated with mAbs as mentioned above. The mixtures were added to A549 cells and incubated at 37°C for 12 h. Cells were washed twice with ice-cold PBS, fixed with 10% NBF, and incubated with an anti-early endosome antigen 1 (EEA1) antibody (RRID: AB_2096811) in PBS containing 10% goat serum and 0.3% Triton X-100. After washed three times with PBS, cells are incubated with an Alexa Fluor 594-labelled secondary antibody (RRID: AB_2338059). Nuclei were stained with DAPI (Beyotime). Fluorescence images were captured by LSM 710 confocal microscope system (Zeiss).

### Data processing and statistics

Flow cytometry data were analysed using FlowJo software v10 (BD). qPCR data were analysed using Bio-Rad CFX Manager 3.1 (Bio-Rad). Confocal images were processed with Zen 2.3 software (Zeiss). Statistical comparisons were performed by Student’s t-test or one-way analysis of variance (ANOVA) and computed with SPSS version 13.0 (SPSS Inc.). A *p-*value < 0.05 was considered statistically significant. Data graphs were constructed using GraphPad Prism 8 (GraphPad). Figures were created using Adobe Illustrator 2021 (Adobe Systems Inc.) or PowerPoint 2013 (Microsoft).

## Results

### Generation of macaque-derived, human-like anti-HAdV55 nAbs

**Figure 1.**
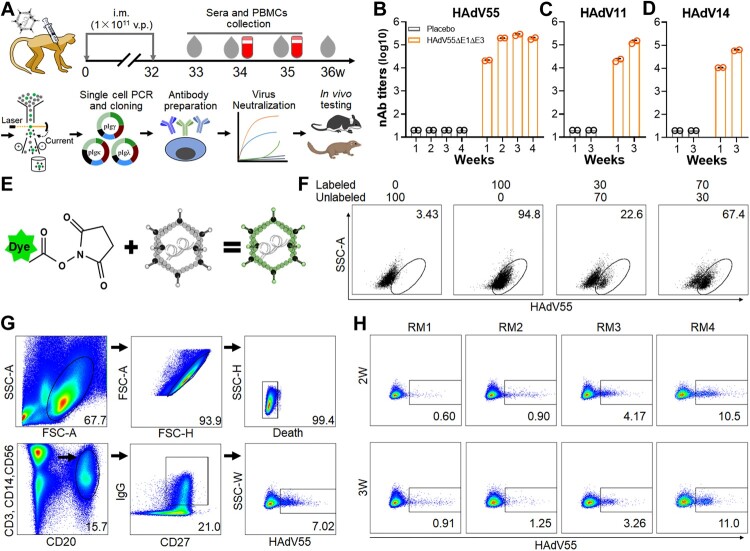
Isolation of HAdV55-specific memory B cells from rhesus macaques immunized with HAdV55ΔE1ΔE3. (A) Schematic diagram of immunization, sample collection, single B cell sorting, antibody gene cloning, and *in vitro* and *in vivo* testing of anti-HAdV55 mAbs. Two macaques (RM3 and RM4) received twice i.m. injections with HAdV55ΔE1ΔE3 at 1 × 10^11^ v.p. per injection at a 32-week interval. Another two macaques (RM1 and RM2) received PBS as a placebo. Sera and PBMCs were collected at the indicated time points and subjected to neutralization assay and memory B cell isolation, respectively. (B-D) nAb responses against HAdV55 (B), HAdV11 (C), and HAdV14 (D) in macaques at 1, 2, 3, or 4 weeks after immunization. HAdV55-SEAP, HAdV11-SEAP or HAdV14-SEAP was incubated with serially diluted macaque sera and was used to infect HEK293 cells. At 24 h after infection, SEAP activities were measured. nAb titres were calculated as the reciprocal of serum dilutions at which 50% of SEAP activities were inhibited. Each data point represents one macaque. The bar indicates the mean value of two macaques, and error bars represent standard deviations (SDs). Shown is one representative result of two repeated assays. (E) Schematic diagram of the labelling of HAdV55ΔE1ΔE3 virions with Alexa Fluor 647 NHS ester. (F) Confirmation of the labelling of HAdV55ΔE1ΔE3. A549 cells were incubated with labelled or unlabelled HAdV55ΔE1ΔE3 separately at 37°C for 2 h, mixed at the indicated ratios, and examined by flow cytometry. Shown is one representative result of two independent experiments. (G) Gating strategy for single memory B cell sorting. HAdV55-specific memory B cells were designated as: CD3^-^, CD14^-^, CD56^-^, CD20^+^, CD27^+^, IgG^+^ and HAdV55^+^. PBMCs (half RM3 and RM4) collected at 2 weeks after immunization were stained with antibodies to the mentioned markers and analysed by flow cytometry. (H) Frequencies of HAdV55-specific memory B cells in macaques at 2 or 3 weeks after immunization. The frequencies of HAdV55-specific memory B cells in total IgG^+^ memory B cells are shown.

During the pursuit of a safe and effective HAdV55 vaccine, we constructed a replication-incompetent HAdV55 of which the E1 and E3 genes were deleted. Because HAdV5 E4 Orf6 protein may form complex with HAdV5 E1B55K more efficiently than with HAdV55 E4 Orf6, which is critical for viral late gene expression and genome replication [44-46], we replaced HAdV55 E4 region with that of HAdV5 to facilitate the propagation of replication-incompetent HAdV55 in HAdV5 E1-expressing HEK293 cells [34]. The resultant HAdV55ΔE1ΔE3 grew to high titres in HEK293 cells. To test the immunogenicity of HAdV55ΔE1ΔE3, we immunized two rhesus macaques (RM3 and RM4) twice at 1 × 10^11^ v.p. per macaque (Figure 1(A)). Another two macaques (RM1 and RM2) receiving PBS were used as controls. At 1 week after the final immunization, we detected high anti-HAdV55 nAbs in immunized macaques. The titres robustly increased at 2 weeks and remained constant thereafter (Figure 1(B)). HAdV55ΔE1ΔE3 immunization also induced anti-HAdV11 and anti-HAdV14 nAbs (Figure 1(C,D)), implying the appearance of both hexon- and fibre-specific antibodies, because HAdV55 shares similar hexon protein with HAdV11 and fibre protein with HAdV14. To isolate HAdV55-specific memory B cells, we labelled HAdV55ΔE1ΔE3 virions with Alexa Fluor 647 via NHS-ester conjugation (Figure 1(E)). Cells bound by the labelled virions could be easily distinguished via flow cytometry (Figure 1(F)). We sorted HAdV55-specific memory B cells using the following markers: CD3^-^, CD14^-^, CD56^-^, CD20^+^, CD27^+^, IgG^+^, and HAdV55^+^ (Figure 1(G)). We observed much higher frequencies of HAdV55-specific memory B cells in immunized macaques than in control macaques (Figure 1(H)), confirming the feasibility of the sorting strategy.

**Figure 2.**
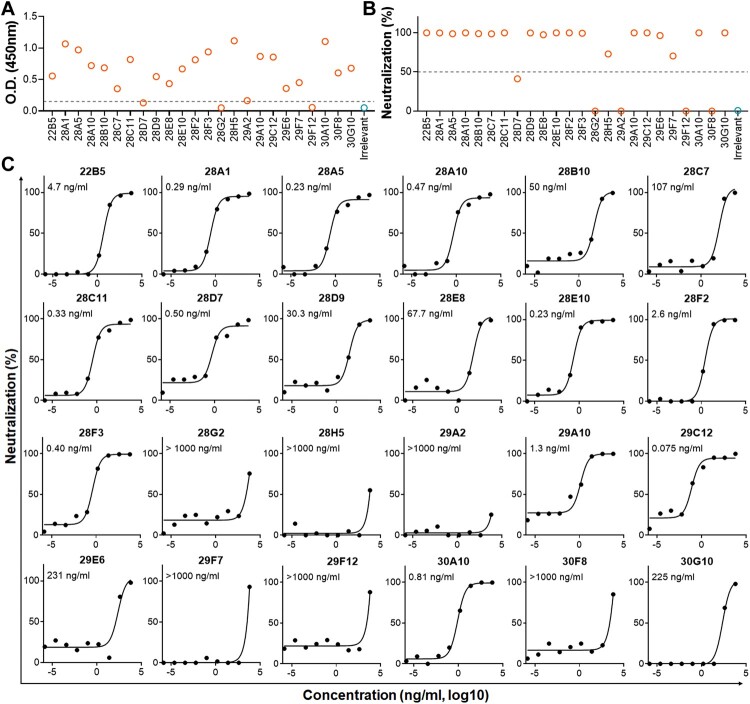
Generation of anti-HAdV55 monoclonal nAbs. (A) Binding activities of each mAb to HAdV55. HEK293T cells were transfected with plasmids encoding the heavy and light chains of each mAb. Culture supernatants were harvested at 3 days after transfection. Binding activities were tested by ELISA using HAdV55 lysates. Shown are the mean OD450 values of 3 replicates. The cutoff value for a positive result is calculated as the mean OD450 value from the irrelevant mAb plus 3 × SD (dashed line). (B) Screening of anti-HAdV55 nAbs. HAdV55-SEAP was incubated with the culture supernatants containing each mAb and was used to infect HEK293 cells. At 24 h after infection, SEAP activities were tested. Neutralizing activity was calculated according to the equation: Neutralization % = (RLU_irrelevant_-RLU_mAb_) / RLU_irrelevant _× 100%. Shown are the mean values from triplicate wells. The dashed line indicates a 50% neutralization. (C) Neutralizing potency of anti-HAdV55nAbs. Each mAb was produced by transfecting Expi293F cells with antibody plasmids and purified using Protein A affinity chromatography. HAdV55-SEAP was incubated with serially diluted mAbs (starting from 6250 ng/ml at 16-fold) and was used to infect HEK293 cells. The IC50 values were calculated as the concentrations at which the SEAP activities were reduced by 50%. All the tests were performed in triplicates and the mean values are shown.

To amplify the antibody-coding sequences from each memory B cell, we performed nested RT–PCR using primers corresponding to rhesus macaque antibody genes [40]. We cloned the segments encoding IgG VH and VL chains into plasmids containing human IgG1 frameworks, and obtained a total of 24 mAbs by transfecting HEK293T cells with each pair of plasmids. Among them, 20 mAbs showed binding activities to HAdV55 (Figure 2(A)), and 19 mAbs appeared to have neutralizing activities (Figure 2(B)). We purified these mAbs and tested their IC50 values. Up to 9 mAbs (28A1, 28A5, 28A10, 28C11, 28D7, 28E10, 28F3, 29C12, and 30A10) had an IC50 value below 1.0 ng/ml. The most potent mAb, 29C12, had an IC50 value as low as 0.075 ng/ml (Figure 2(C)). Notably, the HV chains of 9 and 6 mAbs were derived from germlines IGHV3-71 and IGHV4-4 respectively (Table 1), revealing a preferred usage of IGHV3-71/4-4 among anti-HAdV55 macaque antibodies. IGHV3-71 mAbs tended to pair with IGLV2-11 (3 of 9 mAbs) and IGLV2-23 (4 of 9 mAbs). All three IGHV3-71 mAbs pairing with IGLV2-11 (28A1, 28A5, 28D7) and two of four mAbs pairing with IGLV2-23 (28A10, 28C11) showed high neutralizing potency (IC50 < 1.0 ng/ml) (Figure 2(C) and Table 1). In contrast, IGHV4-4 mAbs appeared to pair with diverse IGKV chains, and all but one mAbs showed only mild or no neutralizing activities. The frequencies of somatic hypermutation (SHM) in HV chains ranged from 5.44% to 16.67%, in LV-kappa chains ranged from 3.94% to 13.26%, and in LV-lambda chains ranged from 1.73% to 8.93%, revealing that most of these chains have been somatically hypermutated. The macaque-human chimeric strategy resulted in similarities with human antibody germline sequences ranging from 86.79% to 96.02% (Table 1). Together, repeated immunization in rhesus macaques elicits high-potent anti-HAdV55 nAbs, which can be conveniently isolated using labelled HAdV55ΔE1ΔE3 virions.

**Table 1.** Information of the mAbs isolated in this study.

mAbs	VH chain						VL chain						Full length identity^d^ %	Targeting sites	IC50 (ng/ml)
	Gene allele^a^	SHM rates^b^	identity^c^ (%)	CDR1 (aa)	CDR2 (aa)	CDR3 (aa)	Gene allele^a^	SHM rates^b^	identity^c^ (%)	CDR1 (aa)	CDR2 (aa)	CDR3 (aa)			
22B5	IGHV3-71	7.48	96.02	GFTFRNYY	LRNKANGGTA	TRGMYSWNNGFDY	IGLV2-23	1.73	95.59	SSDIGYYNA	EVS	SSYAGTGTFYI	95.88	HVR2	4.70
28A1	IGHV3-71	9.52	95.35	GFTFKNYY	IRNKGNGGTA	ARGMYRWNNGFDY	IGLV2-11	2.77	95.59	SSDIGYYDA	DVS	SSYAGSDTFYV	95.43	HVR2	0.29
28A5	IGHV3-71	7.48	96.24	GFTFKNYY	IRNKGNGGTA	ARGMYRWNNGFDY	IGLV2-11	2.77	95.59	SSDIGYYDA	DVS	SSYAGSDTFYV	96.02	HVR2	0.23
28A10	IGHV3-71	6.80	96.24	GFTFKNYY	LRNKANGGTA	TRGMYSWNNGFDY	IGLV2-23	1.73	95.59	SSDIGYYNA	EVS	SSYAGSGTFYI	96.02	HVR2	0.47
28B10	IGHV3-71	6.46	96.89	GFTFGDHY	IRNKANGGTA	GRDLPLHIWTG	IGKV2-30	7.82	94.52	QSLLHSNGNTY	KVS	MQGTHWPFT	96.11	HVR2	50
28C11	IGHV3-71	7.82	95.80	GFTFKNYY	LRNKANGGTA	TRGMYRWNNGFDY	IGLV2-23	4.15	94.27	SSDIGYYNA	EVS	SSYAGSGTFYV	95.29	HVR2, 7	0.33
28D7	IGHV3-71	8.84	95.58	GFTFKNYY	LRNKANGGTA	TRGMYSWNNGFDY	IGLV2-11	3.81	94.71	SSDIGYYNA	DVS	SSYAGSGTFYI	95.29	HVR2, 7	0.50
28D9	IGHV3-71	5.44	96.46	GFTFSNYY	LRNKANGGTA	TRGMYSWNNGFDY	IGLV2-23	3.81	94.71	SSDIGYYNA	EVS	SSYAGSGTFYI	95.88	HVR2	30.3
30G10	IGHV3-71	7.82	97.10	GFTFSDYY	IRNKPNGGTA	ARAYCSWST	IGKV2-29	7.09	93.02	QSLLNSGGKTY	EVS	MQGKQLPFT	95.78	——	225
28C7	IGHV3-69	6.94	96.16	GFTFSDYY	ISNSGGRT	VRDWDY	IGLV1-41	NA^e^	77.53	SSNIGKFSV	NNR	SAWDSSLSGQL	89.85	Fibre	107
28E8	IGHV3-69	7.64	95.50	GFTFIDYY	ISNSGGRT	VRDPTDY	IGLV1-51	2.80	91.59	SSNIGTYY	DNN	GAWDSSLTAQL	94.18	Fibre	67.7
28E10	IGHV3-74	8.33	89.36	GFSFSDYW	ISQPSGR	YCARQGRVHYSGLES	IGKV4-1	6.40	88.70	QSLLYSSNNKNY	RAS	QQYYSAPFT	89.13	HVR1	0.23
28F3	IGHV3-74	10.76	89.83	GFTFSDYW	ISHPSGT	YCVRHRRQFLDGLLSAAGGAVW	IGLV8-61	9.69	92.95	SGSVSSSHH	STE	SLYMGGGISVL	90.86	HVR1, 2, 7	0.40
28G2	IGHV4-4	13.54	92.38	GASMRNYWW	DGNSVTT	ARGYSGYNL	IGKV1-13	13.26	92.06	QNIYTA	GAS	QELYSYPYT	92.27	——	>1000
29A2	IGHV4-4	11.11	92.46	GASISSYWW	NGKSGTT	AKYGSNYGNYGLDS	IGKV1-5	10.39	95.81	QSISNW	KAS	QQYNSYPQVT	93.54	——	>1000
29E6	IGHV4-4	11.81	92.72	GAPISSYWW	NGNSGNT	ARDPVKTVNTGNYFDS	IGKV1-12	4.66	96.26	QGISSW	KAS	QQYNSAART	93.85	Fibre	231
29F7	IGHV4-4	6.94	94.25	GASISSYWW	NGNSGST	ARRPGRYNWNNWFDV	IGKV1D-1	3.94	93.93	QGISSY	YAN	QQGNNNPWT	94.14	Fibre	>1000
30A10	IGHV4-4	14.58	92.04	GGSISGYYW	GGGRGII	ARSPPLDSRDNWFDV	IGKV1-9	NA^e^	75.70	SGHWQL	YTN	QQGKTSPRT	86.79	HVR7	0.81
30F8	IGHV4-4	16.67	91.52	GGSLNNNFW	YVGSGIT	ARDRGSLGLDS	IGKV1-13	7.53	92.99	QVIDTH	NGN	QQANSNPWT	91.99	——	>1000
29C12	IGHV4-38	12.50	92.26	GGSISGYYW	GGGRGVT	ARSPPLDSSDNWFDV	IGKV1-33	7.53	91.59	QDISNY	YTN	QQGKSKPRT	92.04	HVR7	0.075
29F12	IGHV4-38	10.07	91.76	GDSFSGYFW	SGSSGRT	TRTGRRGWYFDY	IGLV3-21	8.93	91.48	NIGSRN	GDN	QVWDSSAKWL	91.67	——	>1000
28F2	IGHV4-39	13.19	91.52	GGSISGNFW	GGGSGVL	ARVAILSYEDEYKKYRREFYFDR	IGLV11-5	5.21	93.51	SDRNVGTKN	YYSDSDK	QVYDSPVVF	92.19	HVR7	2.60
29A10	IGHV4-39	12.15	92.31	GGSISGYYW	AGTRGIT	ARMPPKTIGTTRYRYFDS	IGKV1-9	9.68	91.12	QDINDY	FAN	QQGKSNPYS	91.93	HVR7	1.30
28H5	IGHV4-59	14.74	96.01	GDSMRSNYLY	TYYTGRT	AIGGGSGYLYLEF	IGKV1-9	5.38	92.52	QGINSY	DAN	QQGKSNPYT	94.89	——	>1000

^a^ Germline alleles of the VH and VL of each mAb were identified according to their similarity to human antibody germlines.

^b^ SHM rates of the VH and VL chains were calculated as the percentage of mutated nucleotides according to germline genes (*Macaca mulatta*).

^c^ Similarity of the amino acid (aa) sequences of the VH and VL chains with their corresponding germlines in *homo sapiens*.

^d^ Similarity of the aa sequences of full-length mAbs with their corresponding germlines in *homo sapiens.*

^e^ NA, not analysed.

### Anti-HAdV55 nAbs mainly recognize hexon HVRs and fibre knob

**Figure 3.**
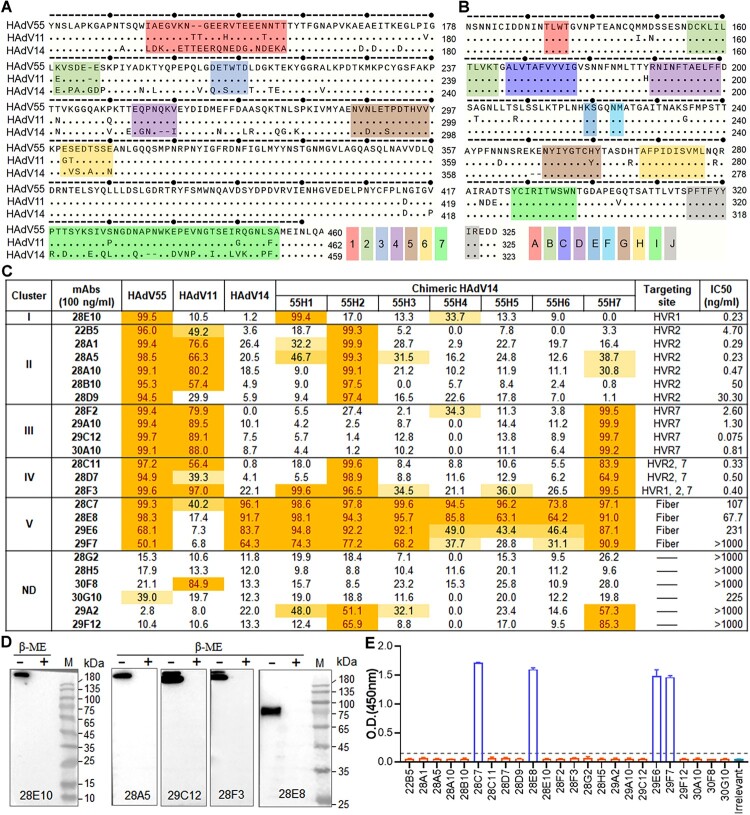
Recognition target profiling of anti-HAdV55 mAbs. (A, B) Amino acid sequence alignment of the hexon HVR (A) and fibre knob (B) of HAdV55, HAdV11, and HAdV14. The 7 HVRs and the β-sheets on fibre knob have been marked with coloured rectangles. (C) Neutralizing activities of the mAbs against HAdV55, HAdV11, HAdV14, and chimeric HAdV14. EGFP-expressing reporter HAdVs were incubated with each mAb at 100 ng/ml and were used to infect A549 cells. At 24 h after infection, EGFP signals were recorded. The neutralizing activity was calculated according to the equation: Neutralization % = (RLU_irrelevant_-RLU_mAb_) / RLU_irrelevant _× 100%. According to the neutralization patterns, the mAbs were classified into 5 clusters, and the putative targeting sites are shown. The IC50 values of the mAbs determined by neutralization assay using HAdV55-SEAP are also listed for reference. ND, not determined. (D) Binding activities of representative mAbs to HAdV55 viral components. HAdV55 lysates were subjected to western-blotting analysis using mAbs 28E10, 28A5, 29C12, 28F3 and 28E8 in the presence (+) or absence (-) of β-ME. The result of mAb 28E10 was derived from a separate membrane. (E) Binding activities of the mAbs to purified HAdV55 fibre knob. ELISA was performed in triplicates and the data are shown as means ± SD.

To dissect the targeting sites of these mAbs, we tested their neutralizing activities against HAdV55, HAdV11, HAdV14, and the seven chimeric HAdV14 of which each hexon HVR has been replaced with the corresponding HVR of HAdV55 (designated as 55H1 to 55H7, respectively) [26]. If a mAb targets hexon, it neutralizes both HAdV55 and HAdV11 because they share similar hexon proteins, unless there are critical mutations inside the targeting sites (Figure 3(A)). Likewise, if a mAb target fibre, it most likely neutralizes HAdV55, HAdV14 and chimeric HAdV14 because they share similar fibre proteins (Figure 3(B)). If a hexon-targeting mAb neutralizes a chimeric HAdV14, it recognizes the particular HAdV55 HVR harboured in that chimeric virus. We identified five clusters of mAbs according to their neutralization patterns. Cluster I contains mAb 28E10 that neutralized HAdV55 and 55H1 but not HAdV11 or HAdV14, implying that this mAb targets hexon HVR1 but the four mutations on HAdV11 HVR1 (T143 and T144 insertions, and R148H and N154T substitutions) abrogate its anti-HAdV11 neutralizing activities (Figure 2(C)). Cluster II contains mAbs 22B5, 28A1, 28A5, 28A10, 28B10, and 28D9, which neutralized HAdV55, 55H2, and mostly also HAdV11 except 28D9, suggesting that these mAbs target hexon HVR2. Cluster III has mAbs 28F2, 29A10, 29C12 and 30A10 that neutralized HAdV55, HAdV11, and 55H7, suggesting that these mAbs target hexon HVR7. Notably, mAbs in cluster IV recognized more than one HVRs. mAbs 28C11 and 28D7 showed neutralizing activities against HAdV55, HAdV11, 55H2 and 55H7, revealing that these mAbs target HVR2 and 7. One mAb, 28F3, was able to recognize HVR1, 2, and 7 because it simultaneously neutralized HAdV55, HAdV11, 55H1, 55H2 and 55H7. Cluster V contains four fibre-targeting mAbs, 28C7, 28E8, 29E6, and 29F7, which neutralized HAdV55, HAdV14 and most of the chimeric HAdV14, but had only mild or no anti-HAdV11 neutralizing activities (Figure 2(C)). Thus, macaque-derived anti-HAdV55 nAbs mainly recognize hexon HVRs and fibre, and hexon HVR2 appears to be frequently recognized.

To confirm the targeting sites of these mAbs, we performed western-blotting analysis of five representative mAbs (28E10, 28A5, 29C12, 28F3, and 28E8) of each cluster using purified HAdV55. We selected these mAbs because they had relatively low IC50 values in their respective cluster. As expected, mAbs 28E10, 28A5, 29C12, and 28F3 bound to hexon trimer (∼300 kDa), whereas mAb 28E8 bound to fibre trimer (∼100 kDa) (Figure 3(D)), consistent to their respective neutralizing patterns (Figure 3(C)). In the presence of β-ME, their binding activities completely disappeared, revealing that the epitopes are all conformation-sensitive. We also tested the binding activities of these mAbs to purified HAdV55 fibre knob protein via ELISA. Four putatively fibre-targeting mAbs, 28C7, 28E8, 29E6, 29F7, but not the hexon-targeting ones, showed significant binding activities (Figure 3(E)). Hence, anti-HAdV55 nAbs mostly recognize conformational epitopes formed by hexon HVR1, 2, 7 or fibre knob.

### Hexon-targeting nAbs confer better protection against HAdV55 infection than fibre-targeting nAbs in hDSG2 transgenic mice

**Figure 4.**
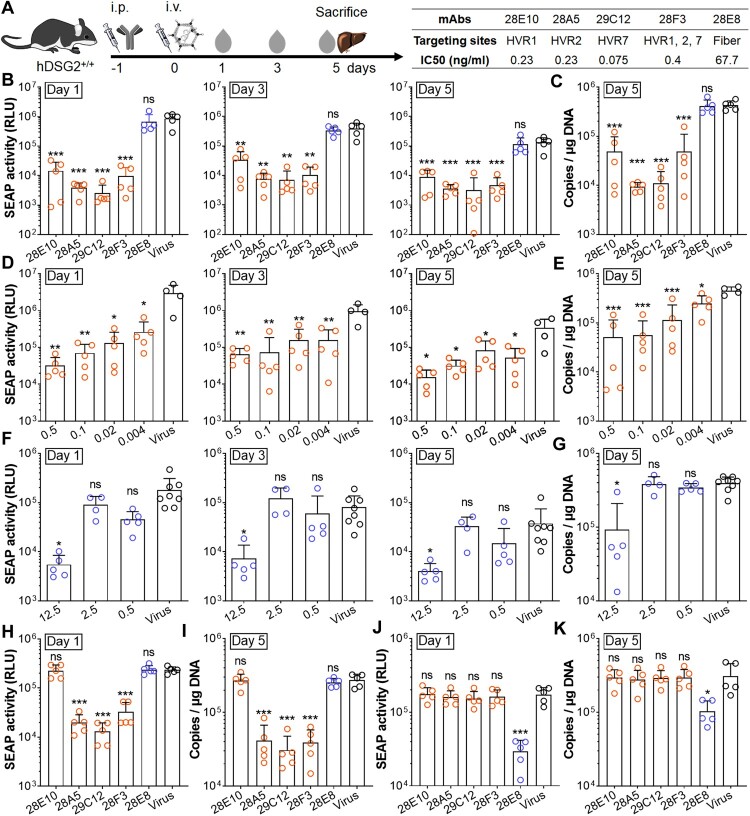
Protective efficacy of anti-HAdV55 nAbs in hDSG2 transgenic mice. (A) Flowchart of the mice experiments. hDSG2 transgenic mice were i.p. injected with each mAb one day before i.v. challenged with HAdV55-SEAP, HAdV14-SEAP, or HAdV11-SEAP at 2 × 10^10^ v.p. per mouse. At 1, 3, or 5 days after challenge, mice sera were collected and subjected to SEAP activity test. At 5 days after challenge, mice were sacrificed and the liver tissues were subjected to viral load test. The mAbs used and their targeting sites and IC50 values (anti-HAdV55) were listed for reference. (B) Serum SEAP activities in mice pre-treated with mAb 28E10, 28A5, 29C12, 28F3, 28E8, or PBS. The results on day 1 (left), 3 (middle), and 5 (right) after challenge are shown. (C) HAdV55 genome copies in the liver of mice pre-treated with each mAb (as described in B) or not. The limit of detection (LOD) is 1 × 10^3^ copies per μg DNA. (D) Serum SEAP activities in mice pre-treated with mAb 29C12 at 0.5, 0.1, 0.02, 0.004 mg/kg, or PBS. The results on day 1 (left), 3 (middle), and 5 (right) after challenge are shown. (E) HAdV55 genome copies in the liver of mice pre-treated with mAb 29C12 (as described in D) or not. (F) Serum SEAP activities in mice pre-treated with mAb 28E8 at 12.5, 2.5, 0.5 mg/kg, or PBS. The results on day 1 (left), 3 (middle), and 5 (right) after challenge are shown. (G) HAdV55 genome copiess in the liver of mice pre-treated with mAb 28E8 (as described in F) or not. (H) Serum SEAP activities in mice pre-treated with mAb 28E10, 28A5, 29C12, 28F3, 28E8, or PBS. The results on day 1 after challenge with HAdV11-SEAP are shown. (I) HAdV11 genome copies in the liver of mice pre-treated with each mAb (as described in H) or not. (J) Serum SEAP activities in mice pre-treated with mAb 28E10, 28A5, 29C12, 28F3, 28E8, or PBS. The results on day 1 after challenge with HAdV14-SEAP are shown. (K) HAdV14 genome copies in the liver of mice pre-treated with each mAb (as described in J) or not. Each data point represents one mouse. The bar indicates the mean of all values, and error bars represent SDs. Shown is one representative result of two independent experiments. Comparison is conducted between each group and the group pre-treated with PBS. Statistical analysis is performed by two-tailed, unpaired Student’s t-test. Significances are marked as: *p < 0.05; **p < 0.01; ***p < 0.001; ns, no significance.

The infection, replication and pathogenicity of HAdV55 in rodents is inefficient due to the lack of cellular receptor and the stringent host range restrictions [20, 21]. Our previously reported hDSG2 transgenic mouse enables an *in vivo* evaluation of anti-HAdV55 nAbs [20]. An i.v. injection with HAdV55-SEAP leads to a substantial elevation of SEAP in the sera. The reduction of serum SEAP activity, therefore, reflects the blocking effects of nAbs against HAdV55 infection. Based on this model, we firstly tested the protective effects of mAbs with distinct targeting sites. We injected hDSG2 mice with mAbs 28E10, 28A5, 29C12, 28F3 or 28E8 at 0.5 mg/kg via an i.p. route. These mAbs represent the most potent ones in their respective cluster. One day later, we challenged the mice with HAdV55-SEAP at 2 × 10^10^ v.p. per mouse via an i.v. injection. At 1, 3, and 5 days after challenge, we measured the serum SEAP activities (Figure 4(A)). Compared to the mock treatment, all the 4 hexon-targeting mAbs, 28E10, 28A5, 29C12, and 28F3, reduced the serum SEAP activities by 50–300 folds on day 1, and 10–50 folds on day 3 and day 5 (Figure 4(B)). In contrast, fibre-targeting mAb 28E8 did not reduce the serum SEAP activities, implying that at this dose mAb 28E8 cannot block HAdV55 infection *in vivo*. We also examined the genome copies of HAdV55 in the liver on day 5. In mice receiving mAbs 28A5 or 29C12, only residual genomes were detected. In those receiving mAbs 28E10 or 28F3, genome copies were also reduced (Figure 4(C)). In line with the unchanged SEAP activities, mAb 28E8 did not reduce HAdV55 genome copies in the liver (Figure 4(C)). Therefore, hexon-targeting nAbs, especially 28A5 and 29C12, are much better than fibre-targeting ones in blocking HAdV55 infection *in vivo*.

To comprehensively dissect the protective effects of mAb 29C12, one of the most potent nAbs in the panel, we tested if a lower dose was still sufficient to block HAdV55 infection. Intriguingly, at both 0.5 and 0.1 mg/kg, mAb 29C12 effectively reduced the serum SEAP activities and the liver viral loads (Figure 4(D,E)). At 0.02 or 0.004 mg/kg, mAb 29C12 also reduced SEAP activities and viral genome copies, albeit to lesser degrees than did by the higher doses. This result further supports the excellent *in vivo* protective efficacy of mAb 29C12.

We next determined whether the fibre-targeting nAb, 28E8, had any protective effects at higher doses. At 2.5 or 0.5 mg/kg, 28E8 showed no inhibition on either serum SEAP activities or liver viral loads (Figure 4(F,G)), revealing no significant protection. At the highest dose we tested, 12.5 mg/kg, 28E8 significantly reduced the serum SEAP activities and liver viral loads. Thus, fibre-targeting nAbs also protect against HAdV55 infection *in vivo*, but only when the dose used is high enough.

To determine whether the anti-HAdV55 nAbs also prevent HAdV11 or HAdV14 infection *in vivo*, we tested the same panel of mAbs in hDSG2 mice which also support HAdV11 and HAdV14 infection. The doses used were as follows: 0.5 mg/kg for hexon-targeting nAbs 28E10, 28A5, 29C12, and 28F3, and 12.5 mg/kg for fibre-targeting nAb 28E8. In line with their *in vitro* neutralizing activities (Figure 3(C)), mAbs 28A5, 29C12, and 28F3 greatly reduced the serum SEAP activities caused by HAdV11 infection, whereas mAb 28E10, which had no anti-HAdV11 neutralizing activities *in vitro*, showed no protection in hDSG2 mice (Figure 4(H)). mAb 28E8, as expected, also showed no significant protection (Figure 4(H)). Consistently, mAbs 28A5, 28F3, and 29C12, but not 28E10 or 28E8, significantly reduced HAdV11 genome copies in the liver (Figure 4(I)). As for HAdV14, no hexon-targeting mAbs showed any protective effects at the tested dose, whereas fibre-targeting mAb 28E8 reduced the serum SEAP activities and liver viral loads (Figure 4(J, K)). Together, a majority of hexon-targeting nAbs confer cross protection against HAdV55 and HAdV11 infection, whereas fibre-targeting nAbs block HAdV55 and HAdV14 infection but a high dose is desirable.

### A single dose of mAb 29C12 prevents HAdV55-caused pneumonia in tree shrews

**Figure 5.**
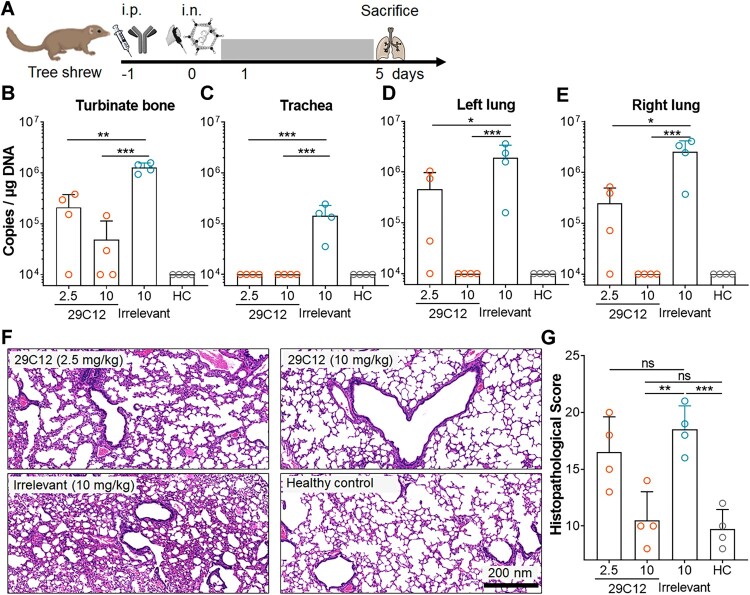
Protective efficacy of mAb 29C12 in tree shrews. (A) Flowchart of the tree shrew experiment. Sixteen tree shrews were divided into four groups. Group 1 and group 2 were i.p. injected with mAb 29C12 (at 10 and 2.5 mg/kg, respectively) one day before i.n. challenged with wild-type HAdV55 at 5 × 10^6^ TCID_50_ per tree shrew. Group 3 received the irrelevant mAb 8D10 at 10 mg/kg and were challenged with HAdV55 similarly. Group 4 received PBS and were used as healthy control. At 5 days after challenge, tree shrews were sacrificed. The turbinate bone, trachea, and lung tissue were harvested and subjected to viral load titration or histological analysis. (B-E) HAdV55 genome copies in the turbinate bone (B), trachea (C), and the left (D) and right (E) lungs. Genome copies were measured by qPCR in triplicates. The LOD is 1 × 10^3^ copies per μg DNA. Each data point represents one tree shrew. The bar indicates the mean of all values, and error bars represent SDs. (F and G) Histopathological analysis of tree shrew lung tissue sections. Lung tissue sections were stained by H&E. Severity of lung injury was scored via a single-blind manner. One representative graph from each group (F) and summary of the histopathological scores (G) are shown. Comparison is conducted among groups. Statistical analysis is performed by one-way analysis of variation (ANOVA). Significances are marked as: *p < 0.05; **p < 0.01; ***p < 0.001; ns, no significance.

To test whether the nAbs have any protective effects against HAdV55-caused diseases, we used tree shrews that have been shown to support HAdV55 replication and pathogenesis [39]. A total of 16 tree shrews were equally divided into 4 groups. Two groups received an i.p. injection of mAb 29C12 at 2.5 or 10 mg/kg, one group received mAb 8D10, an anti-Zika virus mAb, at 10 mg/kg via the same route as an irrelevant control, and a healthy control group received only PBS. One day later, we challenged the tree shrews with wild-type HAdV55 via an i.n. route. At 5 days after challenge, we tested the viral loads in the turbinate bone, trachea, and lung tissues (Figure 5(A)). A single dose of mAb 29C12 effectively inhibited HAdV55 infection (Figure 5(B–E)). In tree shrews receiving 10 mg/kg of mAb 29C12, we detected no viral genomes in the trachea and lung, and only residual genomes in the turbinate bone of 2 of 4 tree shrews. Even at 2.5 mg/kg, this mAb reduced the viral loads in the turbinate bone, trachea and lung as compared to the irrelevant mAb (Figure 5(B–E)), suggesting that mAb 29C12 suppresses HAdV55 infection in the middle and low airways and to a lesser degree in the upper airway. We then examined the lung tissue sections by H&E staining. The tree shrews receiving 10 mg/kg of mAb 29C12 showed no signs of interstitial pneumonia, whereas those receiving 2.5 mg/kg showed moderate interstitial widening. In contrast, the tree shrews receiving the irrelevant mAb showed signs of interstitial pneumonia, including bronchial epithelial hyperplasia, epithelial bronchioles, alveolar cavity dilatation, thickening of interstitium, and inflammatory cell infiltration (Figure 5(F,G)). Thus, mAb 29C12 is able to prevent HAdV55-caused pneumonia in tree shrews.

### Hexon-targeting nAbs arrest HAdV55 in endosome, whereas fibre-targeting nAbs block viral attachment

**Figure 6.**
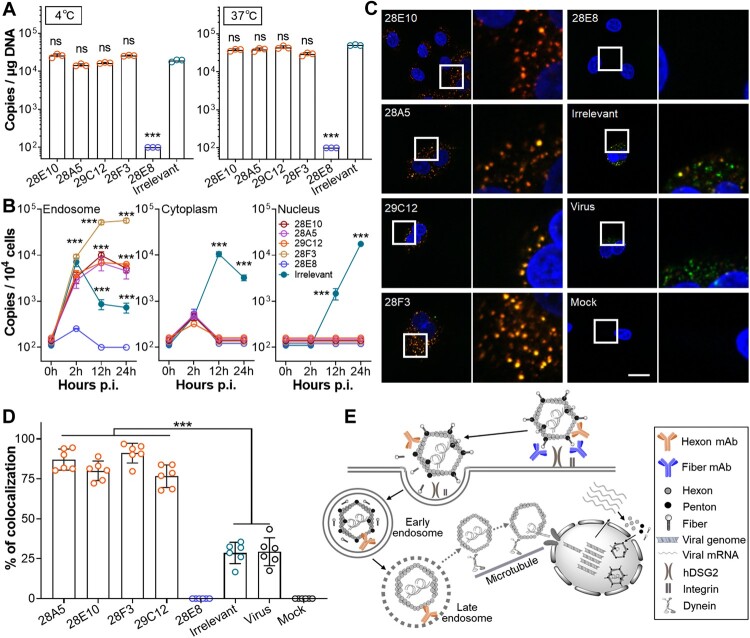
Mechanism of action of anti-HAdV55 nAbs with different targeting sites. (A) Blocking effects of each mAb on the binding and internalization of HAdV55. HAdV55ΔE1ΔE3 was incubated with mAb 28E10, 28A5, 29C12, 28F3, 28E8, or the irrelevant mAb 8D10 at 37°C for 2 h, chilled on ice, and was then used to infect A549 cells at 4°C or 37°C. The bound viruses (4°C, left panel) and internalized viruses (37 °C, right panel) were measured by qPCR. The bar indicates the mean value of triplicates, and error bars represent SDs. Shown is one representative result of three independent experiments. Comparison is conducted between each mAb and mAb 8D10. (B) Blocking effects of each mAb on intracellular transport of HAdV55. HAdV55ΔE1ΔE3 was incubated with mAb 28E10, 28A5, 29C12, 28F3, 28E8, or the irrelevant mAb 8D10 and was used to infect A549 cells. At 0, 2, 12, and 24 h after infection, the endosome, cytoplasm and nucleus were separated and subjected to DNA extraction and qPCR. Each data point represents the mean value of triplicates, and error bars represent SDs. Shown is one representative result of three independent experiments. Comparison is conducted between each mAb and mAb 28E8. The LOD is 1 × 10^2^ copies per μg DNA (A) or per 1 × 10^4^ cells (B). (C and D) Co-localization of HAdV55 viral particles with early endosomes in the presence of anti-HAdV55 mAbs. Alexa Fluor 647-labelled HAdV55ΔE1ΔE3 (green) was incubated with or without mAb 28E10, 28A5, 29C12, 28F3, 28E8, or the irrelevant mAb 8D10 and was used to infect A549 cells. At 12 h after infection, cells were fixed, stained with an anti-EEA1 (red) antibody and DAPI (blue), and observed under laser scanning confocal microscope (C). Scale bar = 20 μm. The frequencies of co-localization were calculated by dividing the total area of overlapped endosomes (yellow) with those of HAdV55 (green and yellow) (D). Each data point represents the value of one version. The bar indicates the mean value of six random versions, and error bars represent SDs. Shown is one representative result from three independent experiments. (E) Possible mechanism of action of the anti-HAdV55 neutralizing antibodies. Fibre-targeting nAbs block the binding of HAdV55 to its primary cellular receptor hDSG2, whereas hexon-targeting ones arrest the viral particles in the early endosome. The symbols of nAbs, viral components and cellular organelles have been indicated. Comparison between two groups (A and B) or among groups (D) were computed by Student’s t-test (unpaired, two-tailed) or one-way ANOVA, respectively. Significances are marked as: ***p < 0.001; ns, no significance.

To dissect the mechanism of action of anti-HAdV55 nAbs, we tested whether these nAbs blocked the binding or entry of HAdV55. We incubated HAdV55 with mAbs 28E10, 28A5, 29C12, 28F3, 28E8, or the irrelevant mAb 8D10 at 1 μg/ml at 37°C for 2 h, chilled on ice, and added the mixtures onto A549 cells. We used this dose because it is sufficient for each mAb to confer neutralizing effects. After incubation at 4°C or 37°C for another 2 h, we measured the viral genomes by qPCR. At 4°C, we detected significant viral genomes in the cells infected in the presence of hexon-targeting nAbs but not in those infected in the presence of fibre-targeting nAb 28E8 (Figure 6(A)). Since incubation at 4°C completely halts the internalization of viral particles [20], this data implies that hexon-targeting nAbs unlikely block the initial viral binding to host cells, whereas fibre-targeting ones completely block this process. Accordingly, at 37°C we detected viral genomes only in the cells infected in the presence of hexon-targeting nAbs but not in those infected in the presence of the fibre-targeting nAb 28E8 (Figure 6(A)). Because endocytosis is active at 37°C, this result confirms that hexon-targeting nAbs, regardless of their targeting HVRs, function at post-binding stages, whereas fibre-targeting nAbs block the initial viral binding to host cells.

We next examined the viral particles in the endosome, cytoplasm, and nucleus of the infected cells at 0, 2, 12, and 24 h after infection. In the endosome of the cells infected in the presence of hexon-targeting nAbs, viral genome copies increased at 2 h, peaked at 12 h, and remained constant or slightly decreased at 24 h, revealing the accumulation of viral particles in the endosome (Figure 6(B)). In the endosome of the cells infected in the presence of fibre-targeting nAb 28E8, viral genomes remained merely detectable throughout the experiment, suggesting that this mAb blocks initial viral binding, and thus subsequent internalization does not occur at all. In the endosome of the cells infected in the presence of the irrelevant mAb 8D10, viral genome copies increased at 2 h but sharply decreased thereafter, reflecting the internalization and subsequent endosomal escape of HAdV55. In the cytoplasm of the cells infected in the presence of either hexon- or fibre-targeting nAbs, only residual, if any, viral genomes were detected (Figure 6(B)). In contrast, in the cytoplasm of the cells infected in the presence of mAb 8D10, viral genomes were merely detectable at 2 h, increased to a peak at 12 h, and decreased thereafter, reflecting the successful viral endosomal escape and subsequent transport to the nucleus. In the nucleus, viral genomes were detectable only in the cells infected in the presence of mAb 8D10 at 12 and 24 h but remained undetectable in the cells infected in the presence of either hexon- or fibre-targeting nAbs (Figure 6(B)). Together, at the tested concentration, hexon-targeting nAbs, regardless of their targeting HVRs, appear to block viral endosomal escape, whereas fibre-targeting nAbs block the initial viral binding, and both ultimately prevent the occurrence of viral genomes in the nucleus.

To further determine the infection stages impacted by each anti-HAdV55 nAb, we infected A549 cells with Alexa Fluor 647-labelled HAdV55ΔE1ΔE3, and stained the early endosome at 12 h after infection. We observed aggregation of viral particles in the endosome of the cells infected in the presence of hexon-targeting nAbs (Figure 6(C,D)), confirming that HAdV55 has been internalized into endosome but fails to escape into cytoplasm (Figure 6(B)). We did not detect any virions in the cells infected in the presence of fibre-targeting nAb 28E8, suggesting that this mAb does prevent viral binding before internalization occurs. In the cells infected in the presence of the irrelevant mAb 8D10, we observed some virions in the cytoplasm or surrounding the nucleus, similar to the cells infected with virus alone, possibly reflecting the successful endosomal escape of HAdV55 and the transport to the nucleus. These data confirm that fibre- and hexon-targeting nAbs function at the initial binding and endosomal escape stages, respectively (Figure 6(E)).

## Discussion

Along with the spreading of highly virulent HAdV55, priority should be given to the exploration of effective antiviral agents. Our successful generation of high-potent nAbs lays the foundation for developing treatments to combat HAdV55-associated severe respiratory diseases. A proportion of nAbs rank the most potent anti-HAdV nAbs reported so far, with IC50 values below 1.0 ng/ml (approximate to be below 6.7 pM). Importantly, mAb 29C12 shows great protection against HAdV55 infection in both hDSG2 transgenic mice and tree shrews. To the best of our knowledge, we are the first to thoroughly test the *in vivo* protective efficacy of anti-HAdV55 nAbs. Our finding that hexon-targeting nAbs confer better protection than fibre-targeting ones supports the notion that protective anti-HAdV antibodies are primarily directed to hexon [35]. The obtained nAbs with distinct targeting sites enable a comprehensive delineation of mechanism of action of anti-HAdV nAbs, and thus expands our understanding about the impacts of host antibody responses on HAdV infection, because previous studies utilized HAdV antisera or a single nAb against one particular HAdV, e.g, mAb 9C12 specific to HAdV5 [29-31, 47].

Recently, macaque-derived mAbs have attracted increasing interest in developing specific antivirals against emerging viruses of epidemic potential [48, 49]. Repeatedly exposure to experimental vaccines promotes antibody maturation via multiple rounds of SHM and antigen selection, leading to antibodies with high affinity [50]. We and other groups have generated macaque-derived nAbs against HIV, Ebola virus, influenza virus and SARS-CoV-2, which have shown high neutralizing potency or cross-neutralizing activities to multiple variants of clinical significance [37, 38, 40, 48, 49]. The utilization of HAdV55ΔE1ΔE3-immunized rhesus macaques may have several advantages: (1) avoiding the screening for blood donors with high titres of nAbs; (2) HAdV55ΔE1ΔE3 is able to mimic the natural HAdV55 infection and thus has good immunogenicity (Figure 1); (3) HAdV55ΔE1ΔE3 carries all the native epitopes targeted by nAbs, and some of them are conserved among multiple pathogenic HAdVs. This is evidenced by the high level of cross-reactive nAbs against HAdV55, HAdV11 and HAdV14 in macaque immune sera (Figure 1). Moreover, we can easily isolate nAbs with high potency from these macaques (Figure 2). The most potent one, mAb 29C12, has an IC50 as low as 0.075 ng/ml (∼ 0.5 pM). Although it is difficult to perform a direct comparison due to different assay settings, the *in vitro* and *in vivo* protective efficacy of our nAbs appear to be at least comparable to or even better than previously reported nAbs such as anti-HAdV55 mAb 9-8-h2 (IC50, 0.6 nM) or anti-HAdV7 HMAb 3-3E (IC50, 0.4 μg/ml, ∼ 2.7 nM) [27, 51]. Thus, it is quite feasible to generate monoclonal nAbs from immunized rhesus macaques.

Until this study, there have been few anti-HAdV nAbs proven to be protective *in vivo*, possibly due to the lack of animal models supporting HAdV infection and pathogenesis [21]. The lack of cellular receptors, as well as the host restriction factors, severely impede the entry and productive replication of HAdVs in commonly used rodents [21]. An earlier study tested an anti-HAdV7 HMAb 3-3E using intracerebrally infected severe combined immune deficiency (SCID) mice [51]. However, an intracerebral infection cannot mimic natural HAdV7 infection, and pre-incubation of HAdV7 with nAbs before injection unlikely reflects the real *in vivo* protective effects [51]. Our utilization of both hDSG2 transgenic mice and tree shrews, therefore, provides in-depth understanding about the *in vivo* protective effects of anti-HAdV55 nAbs. In hDSG2 mice, the inhibitory effects of the nAbs correlated well to their respective *in vitro* neutralizing activities (Figures 2 and 4), which helps to establish a correlation between *in vitro* neutralizing potency and *in vivo* protection efficacy. In tree shrews, a kind of small mammals belonging to the order *Scandentia* and closely related to primates, an i.p. administration of mAb 29C12 dose-dependently prevented HAdV55 infection and the associated lung injury (Figure 5). These two lines of evidence strongly support the preventive and maybe also the therapeutic potential of anti-HAdV55 nAbs.

Previously, we showed that human nAbs elicited by natural HAdV55 infection mainly targeted hexon HVR2, 5, 7 and fibre knob, but epitope preference varied among individuals [26]. In contrast, mouse nAbs elicited by immunization with inactivated HAdV55 predominantly targeted hexon HVR1 and 2 [26]. In HAdV55ΔE1ΔE3-immunized macaques, we isolated nAbs targeting hexon HVR1, 2, 7, both 2 and 7, all of 1, 2, and 7, and fibre knob (Figure 3). We did not isolate HVR5-targeting nAbs, possibly because this kind of nAbs were not provoked due to epitope preference, or their frequencies were too low to be isolated from a small proportion of PBMCs. The modes of antigen exposure and host genetic backgrounds may influence the epitope recognition profile of antibody responses [52, 53]. Natural infection usually starts with a small number of invading viruses, followed by genome replication and progeny virion release. In the context of immunization, however, large amounts of viral particles are simultaneously inoculated. Inactivated HAdV55 is unable to infect host cells and replicate, whereas HAdV55ΔE1ΔE3 might be able to enter host cells and undergo several rounds of viral antigen expression [26]. Consequently, host immune system may encounter different types and quantities of viral antigens, resulting in distinct recognition profiles of antibody responses. Moreover, efficient IgG production relies on cognate interactions between T and B cells. B cells may present different epitopes to CD4^+^ T cells in different hosts, resulting in distinct T and B cell activation [54]. Hence, the dominant epitopes in one host may be not dominant in other hosts.

We did not isolate any penton-targeting mAbs. The successful isolation of specific memory B cells relies on the recognition of the labelled virions by membrane-anchoring B cell receptors (BCR). The accessibility of penton base by BCR may be limited due to the steric hindrance exerted by trimeric fibre protein on the vertex. Consequently, although natural infection or immunization potentially elicits penton-specific antibody responses [26], it is difficult to isolate penton-targeting mAbs using intact virions as probes, and these mAbs unlikely have significant neutralizing activities [26].

Another noteworthy aspect of our study is the successful elucidation of the function mechanism of anti-HAdV55 nAbs. It has been reported that fibre-targeting nAbs block the initial binding of HAdVs into host cells [47, 55]. The detailed neutralizing mechanism of hexon-targeting nAbs, however, remains largely undetermined. Existing evidence show that mAb 9C12, specific for HAdV5 hexon, does not block viral binding, internalization, or endosomal escape, but rather blocks the microtubule-dependent intracellular transport after endosome penetration [29, 47]. This mAb can also bind to tripartite motif containing 21 (TRIM21) by the Fc region, and the latter recruits proteasome and triggers degradation of the viral particles [30]. Our data suggest that hexon-targeting nAbs effectively arrest HAdV55 viral particles in the endosome, which appears to be independent of the particular HVRs they recognize (Figure 6). Because a minor capsid protein, pVI, is critical for the fragmentation of endosome membrane, it is likely that these hexon-targeting nAbs, after binding to the peripenton hexon trimers, inhibit viral disassembly and thereby restrict the release of pVI into endosome [47, 56]. Although the accurate details of anti-HAdV55 nAbs in this process need to be dissected in future studies, the difference between anti-HAdV5 and anti-HAdV55 nAbs may reflect different modes of action of host antibody responses to different HAdVs.

In summary, we generated macaque-derived, human-like anti-HAdV55 nAbs that had great protective efficacy in two animal models, and demonstrated that fibre-targeting nAbs blocked viral attachment whereas hexon-targeting ones mainly blocked viral endosomal escape. These nAbs, especially the hexon-targeting ones, are promising to be further explored as anti-HAdV55 therapeutics. The approach described here can also be used to generate nAbs against other types of HAdVs and other emerging viruses.
